# Insights on the Russian HCV care cascade: minimal HCV treatment for HIV/HCV co-infected PWID in St. Petersburg

**DOI:** 10.1186/s41124-016-0020-x

**Published:** 2016-10-11

**Authors:** Judith I. Tsui, Stephen C. Ko, Evgeny Krupitsky, Dmitry Lioznov, Christine E. Chaisson, Natalia Gnatienko, Jeffrey H. Samet

**Affiliations:** 1 Department of Medicine, Section of General Internal Medicine, University of Washington School of Medicine and Harborview Hospital, 325 9th Ave, Box 359780, Seattle, WA 98104 USA; 2 Department of Global Health, Boston University School of Public Health, 801 Massachusetts Avenue, 3nd Floor, Boston, MA 02118 USA; 3 St. Petersburg Bekhterev Research Psychoneurological Institute, Bekhtereva St., 3, St. Petersburg, 192019 Russian Federation; 4 First St. Petersburg Pavlov State Medical University, Lev Tolstoy St. 6/8, St. Petersburg, 197022 Russian Federation; 5 Pasteur Research Institute of Epidemiology and Microbiology, St. Petersburg, Russian Federation, Mira St. 14, St. Petersburg, 197101 Russian Federation; 6 Data Coordinating Center, Boston University School of Public Health, 85 East Newton Street, 9th Floor, Boston, MA 02118 USA; 7 Department of Medicine, Section of General Internal Medicine, Clinical Addiction Research and Education Unit, Boston Medical Center, 801 Massachusetts Avenue, 2nd Floor, Boston, MA 02118 USA; 8 Department of Medicine, Section of General Internal Medicine, Clinical Addiction Research and Education Unit, Boston University School of Medicine/Boston Medical Center, 801 Massachusetts Avenue, 2nd Floor, Boston, MA 02118 USA; 9 Department of Community Health Sciences, Boston University School of Public Health, 801 Massachusetts Avenue, 2nd Floor, Boston, MA 02118 USA

**Keywords:** HIV, HCV, PWID, Russia

## Abstract

**Background:**

The human immunodeficiency virus (HIV) epidemic in Russia, driven by injection drug use, has seen a steady rise in the past two decades. Hepatitis C virus (HCV) infection is highly prevalent in people who inject drugs (PWID). The study aimed to describe the current frequency of HCV testing and treatment among HIV-infected PWID in St. Petersburg, Russia.

**Methods:**

This study examined baseline data from the “Linking Infectious and Narcology Care” (LINC) and “Russia Alcohol Research Collaboration on HIV/AIDS” (Russia ARCH) studies. Participants included in this analysis were HIV-infected with a history of injection drug use. Descriptive statistics were performed to assess frequency of HCV testing and treatment.

**Results:**

Participants (*n* = 349 [LINC], 207 [Russia ARCH]) had a mean age of 33.8 years (IQR: 31–37) in LINC and 33.0 (IQR: 30–36) in Russia ARCH; 26.6 % (LINC) and 29.0 % (Russia ARCH) were female; 100 % were Caucasian. Nearly all participants had been tested for HCV (98.9 % in LINC, 97.1 % in Russia ARCH). Almost all reported being diagnosed HCV positive (98.9 % in LINC, 97.1 % in Russia ARCH). Only 2.3 % of LINC and 5.0 % of Russia ARCH participants reported ever receiving HCV treatment.

**Conclusions:**

Among these cohorts of HIV-infected PWID in St. Petersburg, Russia, as of 2015 nearly all reported being tested for HCV and testing positive, while only 3.3 % received any HCV treatment. In this new era of effective HCV pharmacotherapy, an enormous chasm in the HCV treatment cascade in Russia exists providing substantial opportunities for curing HCV in HIV-infected Russians with a history of injection drug use.

**Trial registration:**

The studies described were registered with ClinicalTrials.gov through the National Institutes of Health: Linking Infectious and Narcology Care in Russia (LINC) - NCT01612455, registered 1 June 2012, first participant enrolled 3 July 2012; Alcohol’s Impact on Inflammatory Markers in HIV Disease - Russia ARCH Cohort - NCT01614626, registered 25 May 2012, first participant enrolled 15 November 2012.

**Electronic supplementary material:**

The online version of this article (doi:10.1186/s41124-016-0020-x) contains supplementary material, which is available to authorized users.

## Background

Worldwide, hepatitis C virus (HCV) infection is estimated to affect 80 million people [1], placing them at risk for liver cirrhosis, hepatocellular carcinoma, and associated morbidity and mortality [2]. In most parts of the world, HCV transmission occurs through parenteral exposure, including via injection drug use (IDU) [3]. HCV is highly prevalent among people who inject drugs (PWID) through the sharing of contaminated injection paraphernalia [4].

Following the collapse of the former Soviet Union in the 1990s and the Afghan war, Russia experienced increased access to heroin and an upsurge in injection drug use among young adults along with subsequent risk for related viral infections [5]. Harm reduction programs exist in Russia, but are inadequate [6]. Access to needle exchange programs is limited and fear of law enforcement leads to reluctance to carry needles. Currently, opioid agonist treatments are illegal in Russia and thus unavailable for those persons with opioid use disorders [7, 8]. As a result, the prevalence of HIV and HCV among Russian PWID is among the highest in the world. It is estimated that a quarter of PWID in Russia have HIV [9], while the vast majority have HCV [4]. A recent study of HCV prevalence in eight Russian cities reported that PWID in St. Petersburg had the highest prevalence of HCV (90 %) [10]. Recent estimates of HCV prevalence in Russia suggest that 3–4 % of the population is infected [1, 11–13].

With new, direct-acting HCV agents offering attainable sustained virologic response (SVR) or cure, understanding the HCV cascade of care is paramount. The HCV cascade of care describes successive steps of healthcare specific to HCV that result in optimal health outcomes. Areas of the HCV cascade of care include initial screening, confirmatory viral load testing, linkage to care, staging of disease, initiation of therapy, and receipt and adherence to therapy [14]. Deficits along the care continuum have been reported in numerous countries including the U.S., Canada, Australia, and India, particularly among PWID [15–19]. Less is known about gaps in other countries, including Russia, which has one of the largest populations of HIV-infected PWID [20].

Given the confluence of the ongoing Russian epidemic of HCV and HIV co-infection and the new effective HCV treatments, we investigated care for HCV among HIV-infected Russian PWID in St. Petersburg, Russia. Specifically, we evaluated the frequency of HCV screening and treatment, hypothesizing that screening would far exceed treatment in this population.

## Methods

This analysis is a descriptive, observational study on self-reported HCV testing and treatment among HIV-infected Russian PWID. We analyzed data collected from two studies in St. Petersburg, Russia: LINC and Russia ARCH, for which study methods have been previously published [21, 22]. Linking Infectious and Narcology Care (LINC), is a randomized controlled trial testing a peer-led strengths-based case management intervention to link HIV-infected PWID hospitalized at a narcology (addiction) hospital to HIV medical care in St. Petersburg, Russia. Participants were recruited from inpatient wards at the City Addiction Hospital (CAH) in St. Petersburg, Russia from July 2012 through May 2014. Russia Alcohol Research Collaboration on HIV/AIDS (Russia ARCH) is an observational cohort that aims to evaluate the longitudinal association between alcohol consumption and biomarkers of microbial translocation and inflammation. Participants were recruited between November 2012 and June 2015 from clinical HIV and addiction sites, non-clinical sites and snowball sampling in St. Petersburg, Russia. Eligibility criteria for both studies included the following: 1) 18–70 years of age; 2) HIV-infection; 3) having two contacts to assist with follow-up; 4) living within 100 km of St. Petersburg and 5) having a telephone. For LINC, additional criteria included being hospitalized at the narcology hospital, history of injection drug use, and not currently being on antiretroviral therapy (ART) (prior history of ART was not an exclusion). For Russia ARCH being ART-naïve (i.e. never having been on ART) was an eligibility criteria. All study participants provided informed consent and Institutional Review Boards of Boston University Medical Campus and First St. Petersburg Pavlov State Medical University approved the LINC and Russia ARCH studies.

For this analysis, the Russia ARCH sample was limited to participants who were not previously enrolled in the LINC study and who were categorized as PWID (i.e., participant reported at least one of the following: used needles to inject drugs prior to HIV diagnosis or past 30-day IDU). Using responses from the baseline questionnaire, we assessed the following: previous testing of HCV [23], location of HCV testing [23], date of HCV testing [23], physician reported HCV status [23], prior treatment for HCV [23], and date of HCV treatment initiation [23], demographics, HIV risk behaviors [24] and substance use [24–29]. Questions about HCV testing were worded in the following manner: “Have you ever been tested for the hepatitis C virus?”, and “Has a doctor ever told you that you had the hepatitis C virus?” As such, questions did not refer to the specific diagnostic test done (i.e. screening antibody or HCV viral load). Not all questions were asked in both surveys.

## Results

The total sample included 556 HIV-infected Russian adult PWID (*n* = 349 [LINC], 207 [Russia ARCH]). Details of enrollment are presented in Additional file 1: Figure S1 and Additional file 2: Figure S2. In LINC 382 potential participants were assessed, and of those, 349 were found to be eligible and were enrolled and included in the analysis. In Russia ARCH 556 persons were assessed, and of those 365 were found to be eligible and 364 were enrolled. Of those, 90 were also participants in LINC, 13 subsequently disenrolled from the study, and 54 were not known to be injection drug users, and were therefore excluded from this analysis, leaving 207 in the sample. Only 1 % of persons screened for LINC were excluded for current ART use, and 13 % of Russia ARCH persons screened were excluded for past or current ART.

Baseline demographic and other characteristics of each sample, and both samples combined, are shown in Table 1. Participants in these two studies were relatively young, the majority were men, and as is expected for this population, all were Caucasian. The vast majority completed secondary education, and approximately half reported being unemployed. Median time since HIV diagnosis was 7.1 years (IQR = 4-12), and median CD4 cell count was 349 (IQR = 201-550). All in Russia ARCH and the vast majority in LINC were HIV ART-naïve. Current substance use disorders were common in both cohorts (Table 1).

**Table 1:** Baseline demographic characteristics of HIV-infected Russian PWID in LINC (*n* = 349) and Russia ARCH (*n* = 207)

	LINC		Russia ARCH		Total	
Characteristic	No.	(%)	No.	(%)	No.	(%)
Median Age (IQR)	33.8 (31–37)		33.0 (30–36)		33.5 (31–37)	
Sex						
Male	256	73.4	147	71.0	403	72.5
Female	93	26.6	60	29.0	153	27.5
Race						
Caucasian	349	100	207	100	556	100
Other	0	0	0	0	0	0
Education						
Secondary Education or lower	314	90.0	179	86.5	493	88.7
Higher education	35	10.0	28	13.5	63	11.3
Marital Status						
Married or living with partner	105	30.1	96	46.4	201	36.2
Other	244	69.9	111	53.6	355	63.8
Employment						
Full-time	83	23.8	69	33.3	152	27.3
Part-time	41	11.7	22	10.6	63	11.3
Disability	11	3.2	3	1.4	14	2.5
Unemployed	190	54.4	106	51.2	296	53.2
Other (includes retired, homemaker)	24	6.9	7	3.4	31	5.6
Median Monthly Income (IQR)	35000 (20000–50000)		15000 (5000–30000)		28000 (10000–40000)	
Median Years Since HIV Diagnosis (IQR)	6.6 (4–12)		7.4 (4–12)		7.1 (4–12)	
Median CD4 Cell Count (IQR)	*n* = 329 311 (163–492)		*n* = 143 448 (294–639)		*N* = 472 349 (201–550)	
ART (current or past)						
Yes	43	12.3	0	0	43	7.7
No	306	87.7	207	100	513	92.3
Past 12-month drug dependence	*n* = 344					
Yes	326	94.8	127	61.4	453	82.2
No	18	5.2	80	38.6	98	17.8
Past 30-day injection drug use	*n* = 198^a^					
Yes	183	92.4	94	45.4	277	68.4
No	15	7.6	113	54.6	128	31.6
Past 12-month alcohol dependence						
Yes			138	67.0		
No			68	33.0		

*Abbreviations*: *HCV* hepatitis C virus, *HIV* human immunodeficiency virus, *PWID* people who inject drugs, *ART* antiretroviral treatment, *IQR* interquartile range

^a^Available on a smaller sample due to data collection error

Almost all HIV-infected PWID in LINC (345 [98.9 %]) and Russia ARCH (201 [97.1 %]) reported past HCV testing; similarly nearly all participants in LINC (345 [98.9 %]) and Russia ARCH (201 [97.1 %]) reported past physician diagnosis of HCV. In most cases (328 [95.1 %]), the time since HCV diagnosis was ≥ 12 months in LINC. Of 345 reporting HCV testing in LINC, testing occurred in hospitals (193 [55.9 %]), outpatient clinics (107 [31.0 %]), and prisons (37 [10.7 %]) (Table 2). Almost all were HCV treatment-naïve in both LINC (336 [97.4 %]) and Russia ARCH (191 [95.0 %]) (Table 3).

**Table 2:** HCV testing among HIV-infected Russian PWID in LINC (*n* = 349) and Russia ARCH (*n* = 207)

	LINC		Russia ARCH		Total	
Category	No.	(%)	No.	(%)	No.	(%)
HCV testing						
Yes	345	98.9	201	97.1	546	98.2
No	2	0.6	3	1.4	5	0.9
Unknown	2	0.6	3	1.4	5	0.9
HCV Diagnosis (Physician Notified)						
Yes	345	98.9	201	97.1	546	98.2
No	4	1.1	5	2.4	9	1.6
Unknown	0	0	1	0.5	1	0.2
Time since HCV diagnosis	*n* = 345					
≤ 6 months	6	1.7				
6–12 months	11	3.2				
≥ 12 months	328	95.1				
Location of HCV testing	*n* = 345					
Prison	37	10.7				
Outpatient clinic	107	31.0				
Hospital inpatient	193	55.9				
Needle exchange program	1	0.3				
Drug treatment program	6	3.4				
Family planning	2	0.6				
Other	2	0.6				

*Abbreviations*: *HCV* hepatitis C virus, *HIV* human immunodeficiency virus, *PWID* people who inject drugs

**Table 3:** HCV Treatment among HCV/HIV-co-infected Russian PWID in LINC (*n* = 345) and Russia ARCH (*n* = 201)

	LINC		Russia ARCH		Total	
Category	No.	(%)	No.	(%)	No.	(%)
HCV treatment status						
Current HCV treatment	3	0.9	1	0.5	4	0.7
Past HCV treatment	5	1.4	9	4.5	14	2.6
Never treated	336	97.4	191	95.0	527	96.5
Refused to answer	1	0.3	0	0.0	1	0.2
Time of HCV treatment initiation	*n* = 8					
≤ 6 months	1	12.5				
≥ 12 months	7	87.5				
Was SVR achieved			*n* = 10			
Yes			5	50.0		
No			2	20.0		
Don’t know			3	30.0		

*Abbreviations*: *HCV* hepatitis C virus, *HIV* human immunodeficiency virus, *PWID* people who inject drugs, *SVR* sustained virologic response

Among eight reporting prior HCV treatment in LINC, 7 (87.5 %) initiated treatment ≥ 12 months prior. Of 10 with prior HCV treatment in Russia ARCH, 5 (50.0 %) reported achieving SVR and 3 (30.0 %) had unknown outcomes (Table 3). The median age of the 8 HIV-HCV co-infected PWID in LINC receiving HCV treatment was 32.6 (IQR 31.15-34.4), 5 (62.5 %) were never married, all 8 (100 %) were male, 1 (12.5 %) was working part-time, and 5 (62.5 %) completed secondary education or lower. Among ten Russia ARCH participants who reported receiving HCV treatment, the median age was 31.5 (IQR 29-35), 4 (40 %) were married or living with a partner, 6 (60 %) were male, 3 (30 %) were working part-time or full-time, and 9 (90 %) completed secondary education or lower.

## Discussion

This study of two St. Petersburg Russian cohorts of HIV-infected PWID found an enormous gap between testing and receipt of HCV treatment, revealing a “chasm” in the hepatitis C virus (HCV) care cascade. This study found that nearly all participants reported being screened and informed that they had been HCV-infected, suggesting that screening efforts are robust in this population of PWID with HIV. In contrast to nearly ubiquitous rates of screening, few patients (LINC = 2.3 %, Russia ARCH = 5.0 %) reported ever receiving treatment. This points to a chasm with regard to not meeting European Association for the Study of the Liver (EASL) guidelines for prioritizing HCV treatment for PWID [30] and recommendations set forth by Grebely et al. [31]. Based on these results it appears that there is a substantial opportunity to improve care, and corresponding health outcomes, among HIV/HCV co-infected PWID in Russia.

The finding that HCV infection was nearly universal among these HIV-infected PWID in St. Petersburg is consistent with prior literature. Globally, Russia has among the highest burdens of HCV co-infection among PWID with HIV [32]. The HIV epidemic in Russia is primarily driven through parenteral drug use, and in this context, the prevalence of HCV infection, which almost invariably precedes infection with HIV, will be extremely high to omnipresent [33]. Indeed, modeling studies suggest that in countries where HIV is driven by injecting behavior, the prevalence of HCV can be used as a measure of HIV risk [34], and Russia has a high prevalence of HCV among PWID (50–90 %) [4, 10]. Therefore, it is not surprising that nearly all (97–99 %) of these HIV-infected PWID reported being told that they had HCV. However, it is unlikely that in all cases the diagnosis was confirmed with HCV RNA testing. Due to cost constraints, and the fact that patients frequently have to pay out of pocket for these tests, HCV RNA and genotype testing are uncommonly performed in Russia [35]. Given that approximately 25 % of HCV-infected people will spontaneously clear their infection [36], the true prevalence of current HCV infection, rather than past infection with HCV, in this sample was likely lower than reported.

This study demonstrates a large discrepancy between rates of testing and treatment among this population of HIV-infected PWID, many of whom were being treated for their opioid use disorders, which may explain their high testing rates. It appears that efforts to test these high risk patients for HCV infection are successful and thorough. However, despite these high rates of HCV testing, it appears that only a very small fraction (3.3 %) of these HIV-infected PWID can access treatment. In contrast, another study not focused on HIV-infected persons reported lower rates of HCV diagnosis in Russia (40 %), but also low rates of treatment (< 0.1 %) [11]. Similarly, in other countries, such as the U.S., Canada, Australia, and India, it appears that HCV testing efforts often fall short, with many persons, including PWID, being unaware of their infection [17–19, 37–40]. The estimated proportion of persons treated for HCV in the U.S. is also low, 9 % reported in a recent meta-analysis [15], and HIV/HCV co-infected are often not referred for treatment [41]. It may be worth questioning the rationale for such an aggressive HCV testing program, given the limited effort to provide HCV treatment. The potential that awareness of HCV diagnosis positively impacts an individual’s risk behaviors is another rationale for testing; however the evidence for this impact is mixed [38, 42, 43].

A limitation of this study is that HCV status was based on self-report. Also, we did not specify the nature of prior HCV testing in the questionnaire, nor ask about confirmatory viral load or genotype testing. Given the expense of confirmatory HCV viral load testing, it is likely that most participants only had an antibody test. Another limitation is that this study is based on secondary analysis of existing data from two other studies, which included eligibility criteria that participants could not be on ART. Therefore, the sample may not be fully representative of all co-infected PWID in St. Petersburg. The sample might potentially be biased toward patients who are difficult to link to care. However, there were relatively small numbers of participants who were ineligible because of ART use: in LINC 1 %, and in Russia ARCH 13 %. It is likely the sample may be potentially biased toward younger, newly diagnosed HIV-infected PWID with higher CD4 cell counts not meeting criteria for treatment. Russian guidelines at the time of study stated that ART should be initiated for any patients with CD4 cell counts below 350 cells/mm^3^. At baseline, approximately half of the participants in the combined sample had baseline counts below that threshold, and over time we have observed that nearly one quarter of the sample has initiated ART. Another limitation is that we did not ask participants about specific treatments received; therefore, we cannot be sure that the few patients who reported being treated had actually received standard treatment (as opposed to vitamins or other supplements). However, the exact wording of the question (“Have you taken medication to treat hepatitis C, like Interferon and Ribavirin?”) implied anti-HCV treatment that was the standard at the time.

This study was conducted largely before the arrival of direct-acting antiviral (DAA) therapies, when interferon based therapies were used. Of concern is that countries like Russia, which are transitioning from the classification of middle to high income (and thus restricted from generic medications), may be particularly challenged to afford new therapies for HCV. However, the study result speaks to a great need for treatment among co-infected PWID in Russia, particularly given the challenge of meeting WHO’s targets for the goal of “elimination of viral hepatitis as a major public health threat by 2030” [44]. Given that persons who are co-infected with HIV/HCV are at greater risk for having progression of their HCV-related liver disease to cirrhosis and hepatocellular carcinoma, the need is more urgent to address treatment in this population in order to mitigate morbidity and avoid downstream costs [45–47]. Furthermore, treatment of HCV in this PWID population holds the potential to prevent HCV transmission (“treatment as prevention”) [47–49]. Study results also indicate a need for expanded ART as an important initial step in engagement of care.

## Conclusion

Among HIV-infected PWID in St. Petersburg, Russia, nearly all persons reported having been tested and found to have been infected with HCV, yet few (3.3 %) had ever been treated for their HCV infection. As such, the treatment chasm in the Russian HCV cascade of care among these HIV-infected PWID points to the great need for expanded HCV treatment in this population.

## Additional files

Additional file 1: Figure S1. LINC Enrollment. (PDF 230 kb)

Additional file 2: Figure S2. Russia ARCH Enrollment. (PDF 285 kb)

## Abbreviations

ART: Antiretroviral therapy
CAH: City Addiction Hospital
DAA: Direct-acting antiviral
HCV: Hepatitis c virus
HIV: Human immunodeficiency virus
IDU: Injection drug use
IQR: Interquartile range
LINC: Linking Infectious and Narcology Care
PWID: People Who Inject Drugs
Russia ARCH: Russia Alcohol Research Collaboration on HIV/AIDS
SVR: Sustained virologic response
