# Supplementary figures and images for: Insights on the Russian HCV care cascade: minimal HCV treatment for HIV/HCV co-infected PWID in St. Petersburg
Source: Hepatol Med Policy. 2016 Oct 11;1:13. doi: 10.1186/s41124-016-0020-x (PMC5313079; doi:10.1186/s41124-016-0020-x)

**Additional file 1: Figure S1. LINC Enrollment**

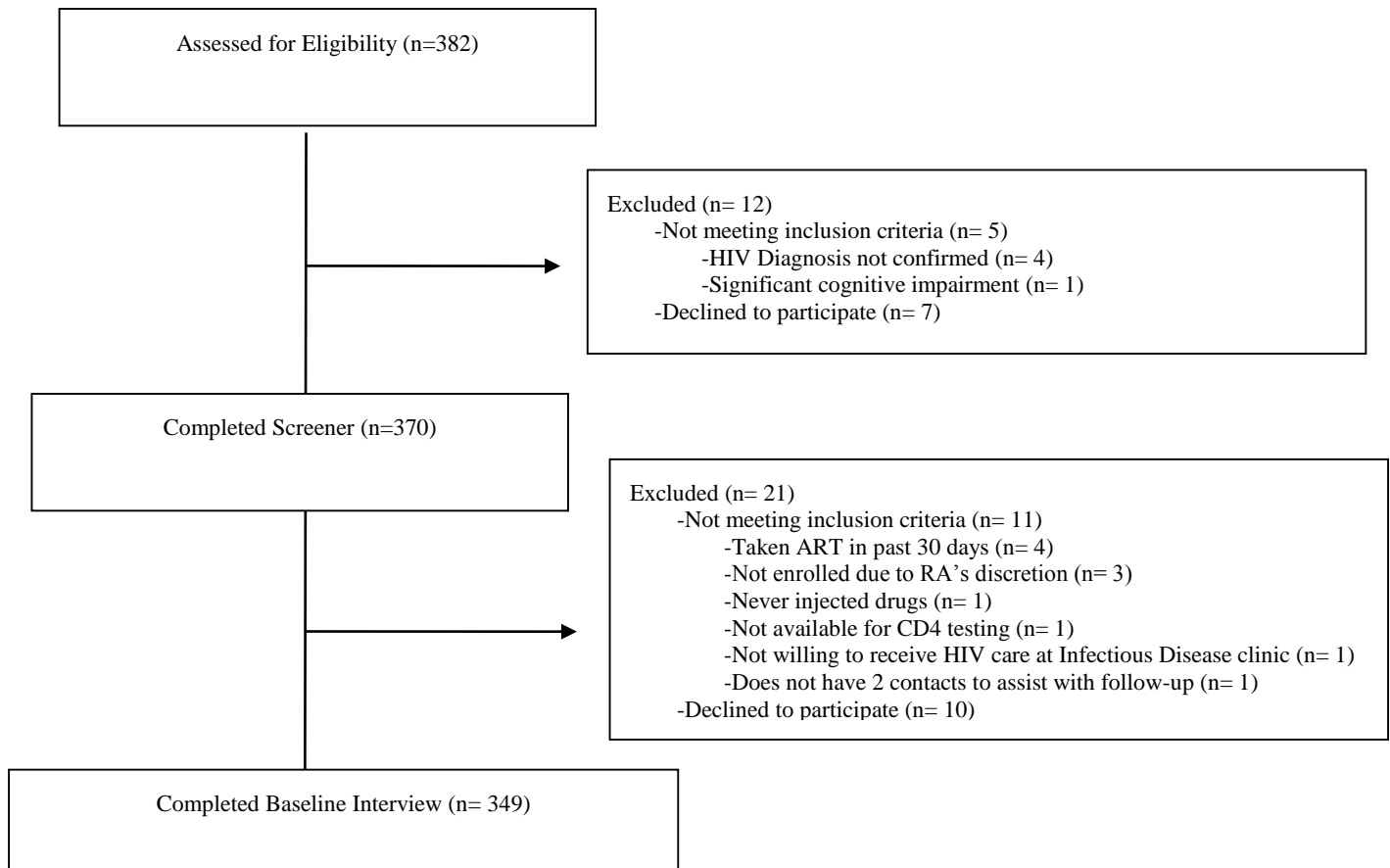

Supplement: Supplementary file 1 — LINC Enrollment. (PDF 230 kb) [file 41124_2016_20_MOESM1_ESM.pdf]

## Additional file 2: Figure S2 Russia ARCH Enrollment

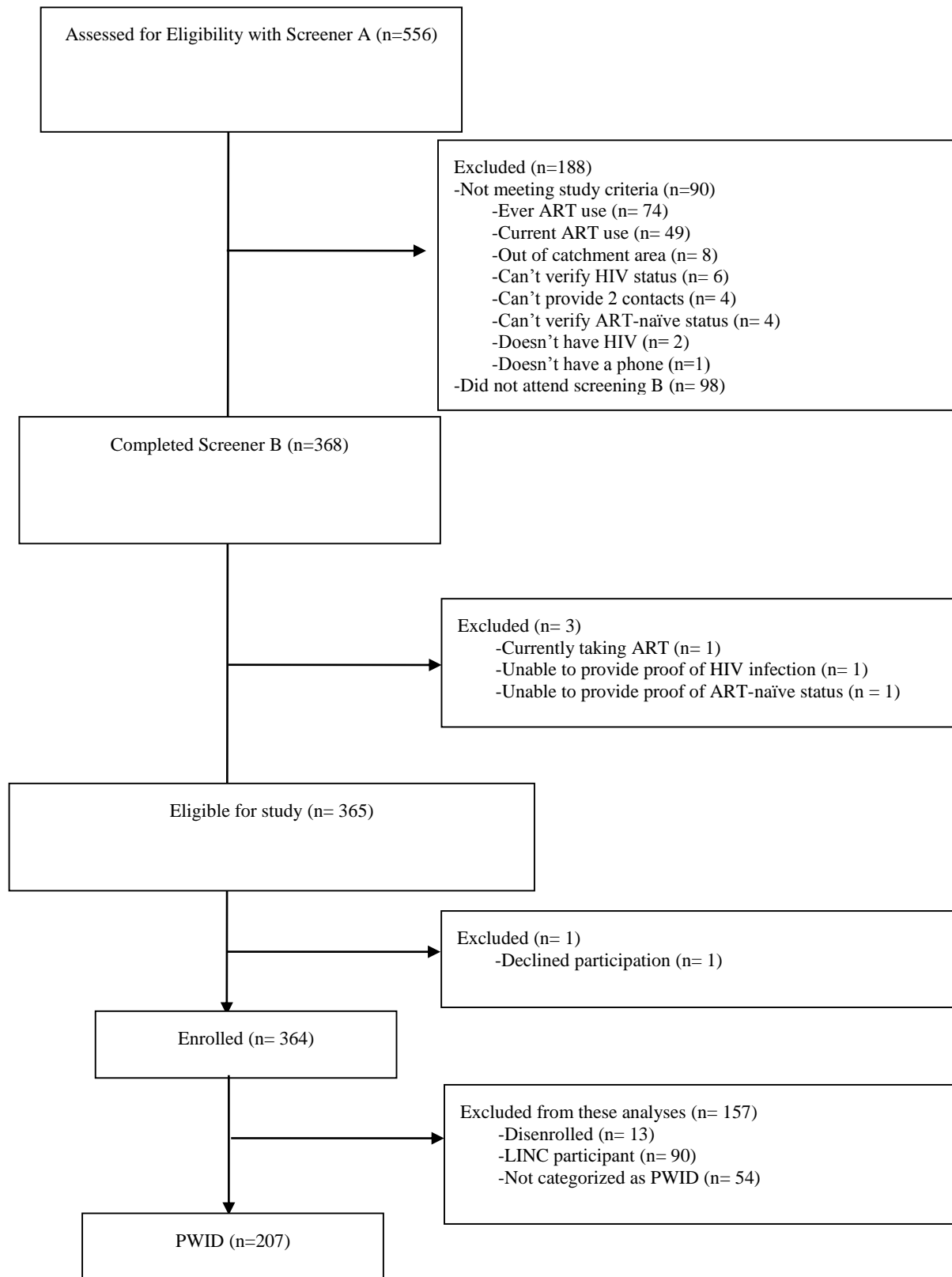

Supplement: Supplementary file 2 — Russia ARCH Enrollment. (PDF 285 kb) [file 41124_2016_20_MOESM2_ESM.pdf]
